# Engineered virus-like particles: paving the way for effective somatic genome editing

**DOI:** 10.1038/s41392-022-01089-6

**Published:** 2022-08-12

**Authors:** Chenya Zhuo, Yu Tao, Mingqiang Li

**Affiliations:** 1 Laboratory of Biomaterials and Translational Medicine, Center for Nanomedicine, The Third Affiliated Hospital, Sun Yat-sen University, Guangzhou, China; 2 Guangdong Provincial Key Laboratory of Liver Disease Research, Guangzhou, China

**Keywords:** Protein delivery, Genetic techniques

A recent publication in *Cell* describes the development and application of engineered virus-like particles (eVLPs) that efficiently package and deliver therapeutic gene-editing proteins, including base editors (BEs) and Cas9 nuclease, with the ability to overcome cargo packaging, release, and localization bottlenecks, representing potentially promising carrier for delivering gene-editing tools of therapeutic interest.^1^

CRISPR technologies have emerged over the past decades and provided new opportunities to advance genetic medicine. A massive race in clinical trials of CRISPR-based genome editing is taking place. Base editing is one of these promising genome editing approaches that can precisely and efficiently achieve targeted single-nucleotide alteration without creating double-stranded DNA breaks, which avoids the potential consequences of other disruptive approaches, such as large indels (insertion-deletions) and chromosomal rearrangement.^2^ Liu’s group has applied this technology to correct pathogenic point mutations in a panel of disease models, indicating its significant therapeutical potential.^1,3^ Finding efficient and safe carriers for delivering BEs to desired tissue targets in vivo remains one of the biggest challenges. Viral vector, such as adeno-associated virus (AAV), is one of the most robust carriers for in vivo BEs delivery.

However, viral delivery itself has several limitations, including the possibility of viral genome integration, and its prolonged expression that may more possibly cause off-target mutations. The gene delivery field has been therefore developing alternative approaches. eVLPs is an emerging approach as it preserves viral vector’s targeting capability but does not hold the aforementioned issues. Previous studies have reported that fusing the desired cargo (protein or mRNA) to the capsid protein of retroviruses, such as gag polyprotein or its homologs, was sufficient for the direct cargo packaging within retroviral particles.^4^ Liu and colleagues therefore made a hypothesis that these retroviral capsid proteins could support efficient BE-eVLPs formation while preserving BE’s activity.^1^

**Fig. 1:**
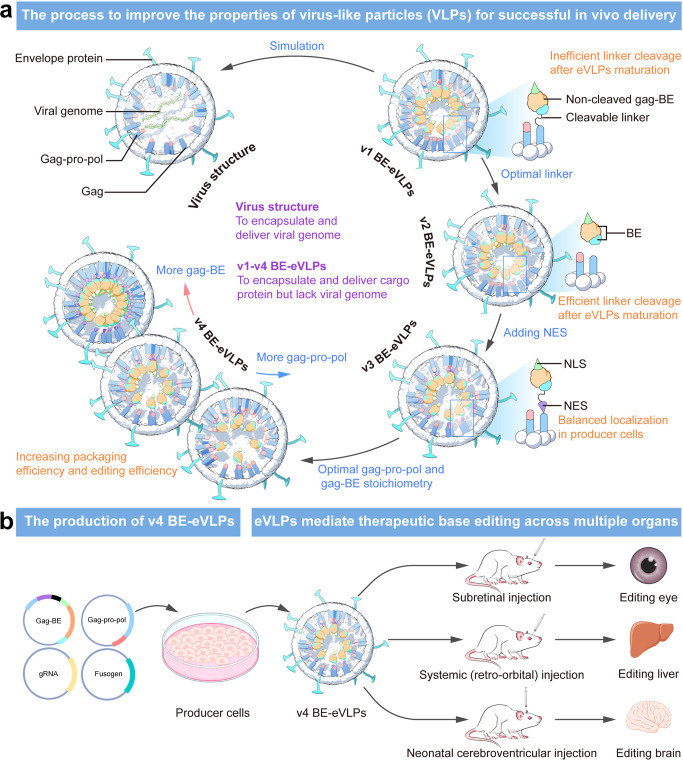
Illustration of structure and engineering approaches of virus-like particles (eVLPs) and the potential applications of v4 BE-eVLPs. **a** The engineering strategies to improve BE-eVLPs for somatic genome editing. Four generations of BE-eVLPs were produced in the study by optimizing cleavable linker, gag–BE fusion localization, and gag–BE:gag-pro-pol stoichiometry. **b** The production of v4 BE-eVLPs and its potential in vivo applications. Adapted with permission.^1^ Copyright 2022, Elsevier

In the initial experiments, to increase the loading capacity and the release efficiency of BE-eVLPs, they made systematic strategies to improve VLP’s architecture and building components.^1^ Four generations of engineered BE-eVLPs were designed as a result (Fig. 1a). In the initial design (v1 BE-eVLPs), ABE8e, an adenine base editor, was fused to a gag polyprotein via a peptide linker, which would be cleaved by the protease after particle maturation. Subsequently, BE-eVLPs were produced by transfecting Gesicle Producer 293T cells with four plasmids expressing gag-ABE8e fusion protein, gag-pro-pol polyprotein, vesicular stomatitis virus G (VSVG) envelope glycoprotein, and single guide RNA (Fig. 1b). However, the editing efficiency of v1 BE-eVLPs did not achieve the expected level at specific loci, indicating that there would be still considerable room to improve BE-eVLP’s activity, such as packaging capacity and editing efficiency.

Liu and colleagues further improved the editing efficiency from three aspects. The gag-ABE8e linker, which could be cleaved by the protease, was first optimized to improve the liberating efficiency of ABE8e RNP after particle maturation. A set of second-generation designs (v2 BE-eVLPs) were constructed. Particularly, v2.4 BE-eVLPs containing protease-cleavable linker (TSTLLMENSS) in the construct exhibited 1.2- to 1.5-fold higher in vitro editing efficiencies compared with the parental v1 design. Further optimization on v2.4 BE-eVLPs was then carried out to generate the third-generation designs, and among these v3 designs, the v3.4 BE-eVLPs, built by adding a 3x nuclear export signal (NES) at the C terminus of gag, promoted the cytoplasmic localization of gag–BE fusion in producer cells, thereby enhancing the gag–BE fusion packaging capacity to improve BE-eVLP’s editing efficiency. Finally, through the optimization of the gag–BE:gag-pro-pol stoichiometry of the v3.4 BE-eVLPs, the fourth-generation v4 BE-eVLPs, which contained 25% gag–BE, showed a 16-fold improvement in cargo protein encapsulation efficiency and 8- to 26- fold increases in editing efficiency. The detailed engineering approach for improving the properties of VLPs to enhance their delivery efficiency is summarized in Fig. 1.

After a series of optimization and in vitro testing, Liu’s group validated their v4 BE-eVLPs in three in vivo base editing applications. Local (neonatal cerebroventricular injection and subretinal injection) as well as systemic (retro-orbital) administration routes have been validated in this study. In all three scenarios, the v4 BE-eVLPs gave the potent, high-efficiency gene-editing performance in the targeted tissues, while maintaining very low off-target effects.

One particularly important advance made by Liu and colleagues is that they strategically engineered the BE-eVLPs approach, enabling efficient base editing in three different organs and minimizing off-target effects and genome integrations, which are the major challenges for somatic genome editing. Currently, AAV is the state-of-the-art for somatic genome editing; yet, some studies have reported the potential for genotoxic integration events after the introduction of AAV in mice, non-human primates, and humans.^5^ The development of these DNA-free eVLPs is of breakthrough significance to overcome this bottleneck in the field. Although the effectiveness of eVLPs-mediated editing has been demonstrated in multiple tissues, the following aspects need to be considered prior to clinical translation. (1) The cargo (e.g., base editors or other CRISPR enzymes) may still cause immune response. (2) During production, eVLPs may carry undesired proteins or mRNAs from the producer cells. Further studies on the cargo purity in eVLPs and these loaded contaminants on transfected cells would warrant for better understanding of this delivery system. (3) Pharmacokinetic studies need to be taken to evaluate eVLPs delivery.

In summary, Liu and colleagues advance the CRISPR delivery by engineering retrovirus eVLPs, and their systematic strategies to improve the performance by modulating the cleavable linker, gag-cargo localization, and stoichiometry of gag-cargo:gag-pro-pol generate an effective and safe eVLPs delivery platform for somatic genome editing. The system is relatively simple and easy to use, allowing other groups to adopt it for their own applications quickly. Their well-designed eVLPs platform is versatile, not limited to BEs delivery; this delivery approach could be also used for the delivery of other CRISPR enzymes or therapeutic proteins, which would have a significant impact in medicine.
